# Advanced and underlying therapeutic strategies in transformed small cell lung cancer

**DOI:** 10.3389/fmed.2026.1865050

**Published:** 2026-08-27

**Authors:** Mingfeng Wei, Zheng Li, Kaiyuan Zhu, Shixiang Guo, Lefei Hu, Fuzhi Yang, Xiaoyu Chen, Xuelin Zhang, Shuai Jiang, Xunxia Zhu, Xiaoyong Shen

**Affiliations:** Shanghai Key Laboratory of Clinical Geriatric Medicine, Affiliated Huadong Hospital, Fudan University, Shanghai, China

**Keywords:** advanced therapy, histologic transformation, lineage plasticity, lung cancer, targeted therapy

## Abstract

Transformed small-cell lung cancer (T-SCLC) is a clinically important form of histologic transformation and a mechanism of acquired resistance in non-small-cell lung cancer (NSCLC). It is associated with poor prognosis, with a median overall survival of only about 9–13 months. This review summarizes recent advances in the mechanisms, diagnosis, monitoring, and treatment of T-SCLC. Repeat biopsy remains the gold standard for confirming histologic transformation, whereas molecular profiling and liquid biopsy may facilitate early detection and longitudinal disease monitoring. Platinum-etoposide remains the most commonly used clinical standard after transformation, but its benefit is typically transient and durable disease control remains uncommon. Continuation of *EGFR* tyrosine kinase inhibitors combined with chemotherapy may prolong progression-free survival in selected patients but has not consistently improved overall survival. Anti-angiogenic therapy, particularly anlotinib, and chemo-immunotherapy have shown encouraging activity in selected patients, while emerging strategies targeting *DLL3*, *MYC*, *SOX2*, and epigenetic regulators may broaden the therapeutic landscape. Prospective studies integrating repeat tissue sampling, comprehensive genomic profiling, biomarker-guided patient stratification, pharmacogenomics, functional drug-sensitivity testing where feasible, and integrated multi-omics approaches are needed to advance molecularly guided and individualized treatment for T-SCLC.

## 1 Introduction

Lung cancer is currently the most common cancers worldwide; according to GLOBOCAN estimates (1), it accounts for 12.4% of all newly diagnosed cancers, making it the leading cause of cancer-related mortality worldwide (2, 3). Pathologically, lung cancer is classified into small cell lung cancer (SCLC, ≈15%) and non-small-cell lung cancer (NSCLC, ≈85%); in the vast majority of cases, the histologic phenotype remains unchanged throughout treatment. In contrast to *de novo* SCLC, T-SCLC is defined as the emergence of SCLC morphology during disease progression after a prior diagnosis of NSCLC, usually following targeted therapy, immunotherapy, or other antitumor treatments. NSCLC is characterized by substantial spatial and temporal heterogeneity, and therapeutic pressure may eliminate sensitive clones while promoting the expansion of resistant subclones. Therefore, T-SCLC may represent either the selection of a pre-existing neuroendocrine-like clone or therapy-induced lineage plasticity. This evolutionary process may also contribute to the short-lived responses observed with platinum-based chemotherapy (4). Importantly, T-SCLC should be distinguished from combined SCLC, which refers to the coexistence of SCLC and NSCLC components within the same tumor at initial diagnosis (5). T-SCLC accounts for 5–14% of all SCLC cases; emergence is linked to *RB1*, *TP53*, and *PI3K*-*AKT* alterations (6, 7).

After histologic transformation, median overall survival (OS) is approximately 9–13 months. Evidence is limited to case series and retrospective cohorts; no standard-of-care exists (8–10). Etoposide plus platinum regimen (EP) chemotherapy is commonly used but efficacy is uncertain. Earlier data suggested targeted agents, immunotherapy, CAR-T, or small molecules may improve prognosis (11). Because early recognition of transformation may influence biopsy timing and treatment selection, surveillance strategies are clinically important. Therefore, this review summarizes current evidence on the mechanisms, diagnostic monitoring, and therapeutic management of T-SCLC. Particular attention is given to genomic alterations, epigenetic regulation, and multi-omics approaches that may support the transition from empiric treatment toward precision management of T-SCLC.

### 1.1 Study design and methodology

This review was based on a structured literature search of PubMed for articles published from 2006 to June 2026. The search strategy combined terms related to NSCLC-to-SCLC transformation, underlying mechanisms, predictive biomarkers, diagnostic surveillance, and therapeutic strategies. Search terms included “non-small-cell lung cancer,” “NSCLC,” “small-cell lung cancer,” “SCLC,” “transformation,” “histologic transformation,” “small-cell transformation,” “lineage plasticity,” “clonal evolution,” “*TP53*,” “*RB1*,” “epigenetic regulation,” “tumor microenvironment,” “predictive biomarker,” “molecular profiling,” “liquid biopsy,” “ctDNA,” “NSE,” “Pro-GRP,” “chemotherapy,” “platinum–etoposide,” “*EGFR-TKI*,” “immune checkpoint inhibitor,” “anti-angiogenic therapy,” “anlotinib,” “*DLL3*,” and “molecular targeted therapy.”

Two investigators independently screened the retrieved literature. Studies were included if they addressed histologic transformation from NSCLC to SCLC and provided relevant information on transformation mechanisms, molecular predictors, diagnostic or surveillance strategies, clinical characteristics, or post-transformation treatment. Reviews, clinical studies, retrospective studies, case series, and case reports were eligible.

**Figure 1:**
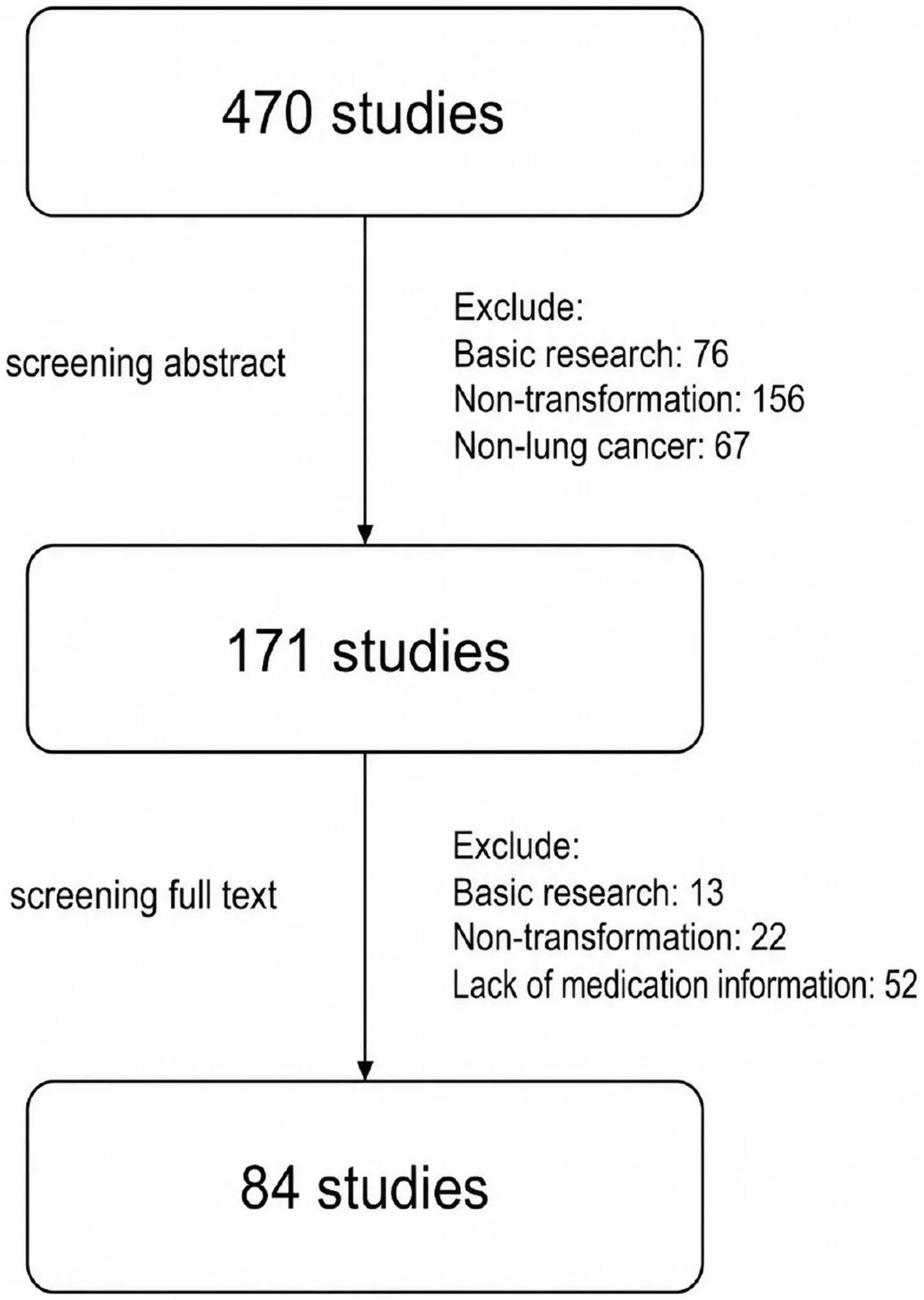
The retrieval flow chart.

A total of 470 potentially relevant articles were initially identified. After excluding duplicate articles, studies with insufficient evidence of histologic transformation, incomplete clinical or treatment information, and inadequate outcome reporting, 84 articles were finally included. The literature selection process is shown in Figure 1. The complete PubMed search strategy is provided in the Supplementary material.

### 1.2 Mechanisms underlying T-SCLC

The mechanisms underlying T-SCLC are complex and involve therapy-induced lineage plasticity, molecular pathway remodeling, and tumor microenvironment changes (12). Current evidence supports two major models of NSCLC-to-SCLC transformation: lineage plasticity and clonal selection (13). Lineage plasticity represents a key biological process enabling NSCLC cells to adopt neuroendocrine-like features characteristic of SCLC (14, 15). The loss of lineage fidelity is frequently linked to concurrent *TP53* and *RB1* inactivation, facilitating phenotypic switching under targeted therapy or immunotherapy pressure (16).

Epigenetic rewiring is an important driver of lineage plasticity. NSCLC-to-SCLC transformation may not depend solely on newly acquired genetic mutations, but may also be mediated by DNA methylation, histone modification, and changes in chromatin accessibility (17, 18). These epigenetic alterations can silence the original NSCLC lineage program while activating neuroendocrine transcriptional states, thereby promoting transition to an SCLC-like phenotype. Regulators such as *EZH2*, LSD1/KDM1A, *DNMTs*, and *HDACs* may contribute to this process by reshaping chromatin structure, regulating lineage-specific transcription factors, and affecting antigen presentation, therapeutic resistance, and immune escape (15, 19).

Remodeling of the tumor microenvironment (TME) may further support the emergence and expansion of T-SCLC clones (12). During transformation, the immune and stromal compartments may become more immunosuppressive, characterized by impaired antigen presentation, reduced cytotoxic T-cell infiltration or T-cell exhaustion, enrichment of suppressive immune cells, abnormal angiogenesis, and extracellular matrix remodeling (20). These changes may help transformed tumor cells evade immune surveillance and adapt to therapeutic pressure, partly explaining the limited efficacy of immune-checkpoint inhibitor monotherapy in T-SCLC (21).

In addition to lineage plasticity, the tumor heterogeneity/clonal selection model may explain some cases (13, 22). A minor SCLC component or neuroendocrine-like subclone may already exist at initial NSCLC diagnosis but remain undetected because of limited biopsy sampling (23). Subsequent therapy may eliminate the dominant NSCLC component, allowing resistant SCLC-like subclones to expand and become predominant (24, 25).

Despite substantial advances in understanding lineage plasticity, epigenetic reprogramming, and tumor microenvironment remodeling, their clinical translation remains limited. Integration of genomic, epigenomic, transcriptomic, and microenvironmental data may therefore be essential for identifying clinically actionable subgroups and treatment vulnerabilities. This gap may reflect the rarity and rapid progression of T-SCLC, marked tumor heterogeneity, limited access to post-transformation tissue, and the lack of prospectively validated biomarkers and targeted treatment strategies.

## 2 Predictive biomarkers and diagnostic surveillance for T-SCLC

One major diagnostic challenge in T-SCLC is distinguishing true histologic transformation from pre-existing, under-sampled combined SCLC at initial diagnosis (26). In some cases, minor neuroendocrine subclones may already be present in NSCLC and subsequently become dominant under therapeutic pressure, leading to apparent transformation (25).

Among molecular biomarkers, *TP53* and *RB1* co-alterations are the best-established molecular risk markers for NSCLC-to-SCLC transformation (27, 28). In *EGFR*-mutant lung adenocarcinoma, concurrent *EGFR*/*TP53*/*RB1* alterations define a particularly high-risk molecular context. Additional genomic features, including whole-genome doubling (WGD) and elevated copy-number variation (CNV) burden, may further refine risk stratification, as they have been associated with earlier and more frequent transformation events (24, 29) (Table 1).

**Table 1:** Recent key studies on RB1/TP53 mutation in NSCLC-to-SCLC histological transformation.

Authors	Year	Cohort	Baseline mutation	Main genetic testing methods	Survival	Reference
Lee et al.	2017	EGFR+LUAD to SCLC (*n* = 21)	Early TP53/RB1 (82%), APOBEC+	NGS	Not reported	(21)
Offin et al.	2019	EGFR+LUAD to SCLC (*n* = 43)	TP53/RB1 (5%), WGD (80%), APOBEC+	NGS + PCR/FISH	TTD:9.5 m (vs36.6 m EGFR only)	(27)
Marcoux et al.	2019	EGFR+NSCLC to SCLC (*n* = 67)	TP53 (79%), RB1 (18%)	NGS + PCR/FISH	OS:10.9 m (after transformation)	(36)
Zhong et al.	2021	NSCLC to SCLC (*n* = 31)	TP53 (90%), RB1 (60%)	NGS	PFS:4.0 m, OS:62.1 m	(30)
Wang et al.	2021	EGFR+LUAD to SCLC (*n* = 32)	TP53 (68%), RB1 (71%)	NGS + PCR/FISH	PFS:3.5 m, OS:9.7 m	(39)
Yu et al.	2022	EGFR+NSCLC to SCLC (*n* = 9)	TP53 (100%), RB1 (71%)	NGS	PFS:3.2 m, OS:8.6 m	(31)
Chen et al.	2023	EGFR+NSCLC to SCLC (*n* = 7)	TP53 (57%), RB1 (43%)	NGS + PCR	PFS:4.7 m	(26)
Tan et al.	2025	EGFR+LUAD to SCLC (*n* = 12)	TP53 (70%), RB1 (30%)	NGS	OS:21 m	(28)

EGFR, epidermal growth factor receptor; LUAD, lung adenocarcinoma; NSCLC, non-small cell lung cancer; SCLC, small cell lung cancer; TP53, tumor protein p53; RB1, retinoblastoma 1; APOBEC, apolipoprotein B mRNA editing enzyme catalytic polypeptide-like; WGD, whole genome duplication; TKI, tyrosine kinase inhibitor; EP, etoposide plus platinum; IO, immunotherapy; NGS, next-generation sequencing; PCR, polymerase chain reaction; FISH, fluorescence in situ hybridization; TTD, time to treatment discontinuation; OS, overall survival; PFS, progression-free survival; m, months; chemo, chemotherapy; anlotinib, a multi-target tyrosine kinase inhibitor.

Serum neuroendocrine markers such as neuron-specific enolase (NSE) and pro-gastrin-releasing peptide (Pro-GRP) may provide supportive clues during disease progression, particularly when their levels increase alongside rapid clinical or radiological progression. However, their limited sensitivity and specificity, as well as potential interference from factors such as hemolysis or renal dysfunction, preclude their use as standalone diagnostic tools (30). Ultimately, repeat tissue biopsy remains the gold standard for confirming transformation, ideally combined with histologic reassessment, neuroendocrine immunohistochemistry, and molecular evidence of clonal evolution (31, 32). Liquid biopsy approaches, including circulating tumor DNA (ctDNA) and circulating tumor cells, together with NGS-based profiling, may facilitate longitudinal monitoring but are currently best applied for risk assessment and early warning rather than definitive diagnosis (33, 34). Emerging third-generation long-read sequencing platforms, such as Oxford Nanopore and PacBio, may further complement conventional NGS by improving the detection of structural variants, methylation patterns, and complex genomic rearrangements, although their application in T-SCLC remains exploratory (35).

## 3 Current therapies

### 3.1 Conventional therapeutic paradigms

T-SCLC biologically resembles *de novo* SCLC but may retain trunk NSCLC driver mutations. Therefore, treatment after transformation generally follows SCLC-based chemotherapy, while retained actionable alterations may also be considered (Table 2).

**Table 2:** Recent studies on therapies after histological transformation from NSCLC to SCLC.

Authors	Year	Patients	Proportion of TKI treatment before transformation	Time to transformation	Chemotherapy after Transformation	TKIs after transformation	Immunotherapy after transformation	Other targeted	Survival	Reference
Norkowski et al.	2013	9	2	49 m	7	not available	not available	not available	16 m	(25)
Ferrer et al.	2019	61	39	16 m	52	2	1	0	9 m	(38)
Marcoux et al.	2019	58	58	17.8 m	56	34	17	no	10.9 m	(36)
Wang et al.	2021	29	25	27.5 m	27	18	6	18 for Anti-angiogenic agents	14.8 m	(55)
Wang et al.	2021	32	32	17 m	30	0	3	5 for Anti-angiogenic	9.7 m	(39)
Zhong et al.	2021	31	25	33 m	30	3	3	not available	11 m	(30)
Saalfeld et al.	2024	47	47	19 m	47	10	20	0	11 m	(20)
Wang et al.	2025	140	40	20.0 m	3	not available	not available	not available	11.0	(37)
Chen et al.	2025	27	27	16.1 m	12	10	5	not available	39.5 m	(62)
Catania et al.	2025	25	24	19 patients >12 m; 6 patients ≤12 m	12	5	7	none specified	mPFS 2 m; mOS 9 m	(7)

NSCLC, non-small cell lung cancer; SCLC, small cell lung cancer.

### 3.2 Chemotherapy

#### 3.2.1 EP regimen: etoposide plus platinum

Both NCCN (Small-cell Lung Cancer) guidelines and SEOM (Spanish Society of Medical Oncology) clinical guidelines platinum–etoposide as a first-line regimen for SCLC. This regimen is widely used in both limited-stage and extensive-stage disease (36, 37).

Despite a high recurrence rate, EP remains the most commonly used regimen with the largest available clinical experience in T-SCLC (38). T-SCLC, particularly when derived from adenocarcinoma, initially shows EP sensitivity comparable to that of primary SCLC (about 80% ORR), yet long-term outcomes are inferior (39). This disparity likely reflects retention of canonical driver mutations (*EGFR*, *KRAS*, *ALK*) in T-SCLC, whereas *de novo* SCLC lacks these alterations (11). In a 2025 study by Wang et al., all cases of T-SCLC arose from *EGFR*-mutant adenocarcinoma; first-line EP yielded median progression-free survival (mPFS) 3 month and mOS 5.7 month, mirroring historical data but inferior to de novo SCLC. Adding *EGFR-TKI* did not extend OS, indicating a biologic shift post-transformation (39). In a retrospective study by Marcous et al., patients with *EGFR*-mutant adenocarcinoma who had transformed to SCLC received first-line EP chemotherapy, achieving an ORR of 54% and a mPFS of 3.4 months (38). Ferrer reported EP response rates of 45% in *EGFR*-mutant T-SCLC and 40% in *EGFR*-wild-type T-SCLC (40), Wang’s multicenter retrospective analysis of *EGFR*-mutant adenocarcinoma patients who transformed to SCLC after *EGFR-TKI* showed EP remained the most used regimen, yielding an ORR of 44.4% and mPFS of 3.5 months (41).

Beyond *EGFR*-mutant cases, rare transformation events have been documented in *ALK* or *ROS1*-rearranged lung cancers. Lin et al. described a *ROS1*-fusion-positive adenocarcinoma that switched to SCLC, lost responsiveness to *ROS1*-directed therapy, and progressed fatally within weeks of initiating chemotherapy (42). In Hobeika’s retrospective series of eight *ALK*-rearranged patients who transformed, only three achieved a partial response to platinum-based chemotherapy combined with an *ALK* inhibitor, whereas the remainder died within weeks (43). Owing to the paucity of cases, the efficacy of EP-centered regimens in non-*EGFR*-driven transformation remains undefined, underscoring the urgent need for prospective investigations tailored to these molecular subgroups (43, 44).

Although other forms of histological transformation, such as transformation to lung squamous-cell carcinoma, have occasionally been reported, they are beyond the scope of this review. The present review focuses on transformation to SCLC because of its clinical relevance, poor prognosis, and distinct therapeutic challenges (45–47). Although EP remains widely used in T-SCLC and achieves a high ORR, disease progresses rapidly after transformation; progression-free survival is only a few months, so most patients progress within months and switch to second-line therapy (39). Therefore, multiple trials have combined first-line chemotherapy with radiotherapy (48), targeted agents (4), immunotherapy (38, 48), or novel small-molecule inhibitors (49) to prolong PFS and improve ORR, and some of the studies yielded promising results, which are presented in the corresponding sections (25).

#### 3.2.2 Taxanes

Beyond EP, taxanes have been reported for T-SCLC. They stabilize microtubules, halt mitosis, and trigger apoptosis (50); additionally, they activate T-cell–mediated immunity, which may contribute to their antitumor activity (51).

In the retrospective cohort of Marcous et al., *EGFR*-mutant T-SCLC treated first-line with paclitaxel achieved a 50% response rate and 2.7-month mPFS, outperforming the 20–30% response rate historically seen in *de novo* SCLC (38). The enhanced sensitivity of T-SCLC to taxanes is presumed to result from *EGFR* mutation-related biological vulnerability, partial retention of adenocarcinoma features that confer better taxane response, and the agents’ concurrent immunomodulatory effects that may potentiate antitumor immunity (52). Yet, in the same Marcous cohort, the response rate to docetaxel was 0%, indicating that taxanes are best reserved for *EGFR*-mutant T-SCLC, patients who are intolerant or refractory to EP, or those requiring rapid tumor debulking, serving as a second- or later-line option after EP failure (38, 52).

Much of the evidence supporting platinum–etoposide and taxanes in T-SCLC is derived from retrospective studies conducted in earlier therapeutic eras. Therefore, although objective responses are frequently observed, progression-free survival is generally limited to only a few months, and these responses should not be interpreted as durable clinical benefit.

### 3.3 Radiotherapy

In *de novo* SCLC, radiotherapy provides clear clinical benefits: early concurrent chemo-radiation in limited-stage disease doubles 2-year survival without added severe toxicity, and even selected extensive-stage patients benefit from consolidative thoracic radiotherapy (cTRT) after chemotherapy (53, 54).

However, local radiotherapy is not typically used as first-line monotherapy in T-SCLC and is usually combined with chemotherapy (25). Previous studies showed that T-SCLC patients given local radiotherapy during chemotherapy achieved significantly longer OS than those without irradiation (15.5 vs. 13.9 months, *p* = 0.045), and multivariate analysis identified local radiotherapy as an independent favorable factor for post-transformation survival (HR 0.28, 95% CI 0.08–0.97, *p* = 0.044), suggesting that local radiotherapy may be considered as part of multimodal management in selected patients of multimodal management (55).

## 4 Targeted therapy strategies

Comprehensive molecular profiling may help identify retained driver alterations, newly acquired genomic dependencies, and epigenetic vulnerabilities that could guide treatment selection after transformation. *EGFR-TKI* targeted therapy is generally ineffective in de novo SCLC, because SCLC is mainly driven by *TP53*/*RB1* inactivation rather than canonical receptor tyrosine kinase drivers such as *EGFR* or *ALK* (23, 56). T-SCLC has a more complex molecular profile: it may retain trunk driver alterations from the original NSCLC clone, while acquiring SCLC-like features such as *TP53*/*RB1* loss and reduced *EGFR* signaling activity (27, 31, 32, 41). Therefore, prior *EGFR-TKIs* often lose efficacy after transformation, and treatment strategies should shift toward SCLC-oriented regimens and emerging molecular targets (57). Genomic alterations such as *PTEN* loss and *NOTCH1* inactivation, together with targets such as *DLL3* (Delta-like ligand 3), provide a rationale for novel targeted or combination therapies in T-SCLC (58, 59).

### 4.1 Continuation of *EGFR-TKI* therapy and combination strategies

Although a subset of T-SCLC patients retain the baseline *EGFR* mutation, retrospective series consistently report negligible objective response rates and abbreviated progression-free survival when *EGFR-TKI* monotherapy is continued after transformation (60). In Yu et al.’s observation, 8 of 9 T-SCLC patients retained the original EGFR mutation (predominantly exon 19 deletion), yet *EGFR-TKI* monotherapy or combination yielded a median PFS of only 3.2 months—far below the response observed during their prior NSCLC phase (32). Likewise, Xu et al.’s systematic review of 72 T-SCLC cases showed that, although 87% retained *EGFR* mutations, TKI monotherapy produced an ORR of only 50% and a median PFS of 3.5 months-markedly inferior to the response seen during the NSCLC phase (57). This attenuation is thought to reflect post-transformation down-regulation of *EGFR* and its downstream signaling proteins, reducing oncogenic reliance on the receptor; the frequent disappearance of the T790M resistance mutation further indicates that the *EGFR* axis is “bypassed” during SCLC conversion. This phenotype is especially common among never-smoker females (40). Importantly, biallelic inactivation of *TP53* and *RB1* constitutes the molecular signature of SCLC, a feature recapitulated in T-SCLC. Yu et al. (32) documented *TP53* mutations in 100% of transformation cases and *RB1* loss in 71%, a genomic profile virtually consistent with *de novo* SCLC. These findings suggest that, post-transformation, tumor cells abandon *EGFR* oncogene addiction and instead rely on neuroendocrine programs or alternative survival axes, such as *MYC* amplification, *NOTCH* silencing, or *PI3K*/*AKT* activation—for proliferation and resilience (41, 61).

Although *EGFR-TKI* efficacy declines sharply after histologic transformation, pooled data indicate that chemotherapy plus continued TKI yields superior objective response rates and progression-free survival compared with either modality alone, yet fails to confer a statistically significant overall survival advantage (28).

In Wang et al.’s cohort of 29 *EGFR*-mutant adenocarcinoma patients who transformed to SCLC, those receiving chemotherapy plus *EGFR-TKI* achieved an objective response rate of 43.8% versus 37.5% with chemotherapy alone; combination therapy also significantly prolonged mPFS (5.2 months vs. 3.0 months, *p* = 0.014), yet median overall survival remained comparable between the two arms (14.8 months vs. 13.0 months, *p* = 0.474) (55). In Chen et al.’s cohort of 27 *EGFR*-mutant adenocarcinoma patients who transformed to SCLC, the chemotherapy-plus-*EGFR*-*TKI* arm achieved an ORR of 44.4% and mPFS of 7.2 months, surpassing the chemotherapy-alone arm (16.7% and 3.7 months); nevertheless, median OS was virtually identical (38.9 vs. 34.4 months, *p* = 0.92) (62). Additionally, Offin et al. identified patients harboring concomitant *EGFR*/*TP53*/*RB1* mutations as a high-risk cohort for SCLC transformation, with an incidence reaching 18% (28), whereas the conversion rate among individuals bearing *EGFR* mutation alone ranges from only 3 to 14% (63). These high-risk individuals frequently undergo histologic transformation soon after *EGFR-TKI* initiation. Retained *EGFR* mutation sustains partial TKI sensitivity, while the emergent *TP53*/*RB1* SCLC component remains sensitive to chemotherapy; consequently, dual-agent regimens yield superior disease control versus monotherapy, yet fail to confer a significant OS advantage (8).

### 4.2 Anti-angiogenic agents

Anlotinib is an oral, multi-target tyrosine-kinase inhibitor that blocks vascular endothelial growth factor receptors (*VEGFRs*), platelet-derived growth factor receptors (*PDGFRs*), and additional kinases, thereby inhibiting angiogenesis and tumor cell proliferation (64). When combined with cytotoxic agents, anlotinib suppresses neovascularization, induces transient vascular “normalization,” lowers interstitial fluid pressure, and augments both intratumoral distribution and retention of concurrent chemotherapeutic or targeted drugs, thereby elevating effective intralesional concentrations and achieving synergistic cytotoxicity (65).

In third-line or later therapy, as well as in chemo-immunotherapy combinations and maintenance settings, anlotinib has shown preliminary activity in retrospective or selected clinical settings and an acceptable safety profile, and is among the few targeted agents reported to extend overall survival in T-SCLC (41). In a separate cohort of 18 individuals, those treated with anlotinib—either as monotherapy or in combination with chemotherapy, *EGFR-TKI*, or immunotherapy—achieved a median OS of 15.1 months, compared with only 4.3 months in patients who did not receive anti-angiogenic therapy (*p* < 0.001, hazard ratio = 0.04) (55). Moreover, the SCLC subset of Cheng’s study confirmed that anlotinib as third-line-or-beyond therapy significantly prolonged mPFS (4.3 vs. 0.7 months; HR 0.19, *p* < 0.0001) and median OS (7.3 vs. 4.9 months). Consequently, the Chinese Society of Clinical Oncology (CSCO) guidelines have incorporated anlotinib for relapsed SCLC after ≥ two prior lines, providing guideline-level support for its use in transformed disease; larger patient series are awaited to consolidate its therapeutic role in this setting (64).

### 4.3 Investigational molecular targets and early-phase therapeutics

Owing to the rarity of T-SCLC, most novel targeted agents have been evaluated exclusively in de-novo cohorts; nevertheless, the near-identical genomic landscape and neuroendocrine biology of the two entities justify extrapolating these early-phase data to transformed cases, thereby expanding the therapeutic armamentarium for T-SCLC (56).

#### 4.3.1 ALK-TKI

Transformation to SCLC among *ALK*-positive patients is uncommon; however, post-transformation specimens consistently reveal concurrent *RB1* and *TP53* inactivation, underscoring these alterations as critical molecular drivers of histologic switching (43, 66). The likelihood of transformation also varies with *ALK-TKI* generation. Second-generation agents—exemplified by alectinib—exert more potent and selective *ALK* suppression than crizotinib, thereby intensifying selective pressure and predisposing tumors to SCLC transformation (67). A study documented only two patients transformed while on crizotinib, whereas the remaining seven conversions occurred during second-generation TKI therapy (68).

Therapeutic sequencing in *ALK*-rearranged transformation is challenging. In published cases, patients generally received standard SCLC chemotherapy (EP/EC) as first-line, with ORRs of 50–66% but median PFS of only 3–4 months. Subsequent chemo-immunotherapy regimens produce even shorter durable benefit, and median OS ranges 6–12 months (comparable to the 8–12 months reported for *EGFR*-driven conversions). The transformed tumors retain the original *ALK* fusion, but remain virtually refractory to second-generation *ALK-TKIs*, underscoring “oncogene retention with phenotypic escape” as a shared resistance mechanism (44). Nevertheless, case-based evidence suggests that *ALK* inhibition may retain some activity in selected SCLC or transformed-SCLC tumors harboring persistent *ALK* rearrangement, although the durability of response and the underlying biological mechanism remain uncertain (69).

#### 4.3.2 *PI3K*/*AKT*/mTOR axis inhibitors

The *PI3K*–*AKT–mTOR* axis constitutes a central intracellular signaling nexus that regulates cell growth, survival, and metabolism; its constitutive activation is frequently documented in both de-novo and transformation SCLC (70). Pre-clinical and early-phase clinical data demonstrate that *PI3K*, *AKT*, and *mTOR* inhibitors—alone or in rational combinations—exert potent antitumor activity against SCLC; adherent variant SCLC cell lines exhibit nanomolar sensitivity (IC₅₀ ≈ 100 nM) to dual *PI3K*/*mTOR* blockade. Notably, the higher the baseline pathway activation, the greater the chemoresistance, yet the more exquisite the dependency on *PI3K*/*mTOR* inhibition (71). No clinical trial has yet evaluated *PI3K*/*AKT*/mTOR inhibitors in T-SCLC; nevertheless, the near-identical genomic landscapes of de-novo and transformed disease provide a strong biological rationale for their investigation in this setting.

#### 4.3.3 Additional potential therapeutic targets

*MYC* amplification enhances tumor aggressiveness and chemoresistance and may facilitate the transition toward a neuroendocrine-low SCLC phenotype (22, 72). Offin et al. documented *MYC* amplification in 11.6% of *EGFR*/*RB1*/*TP53* triple-mutant T-SCLC cases (28). Gardner et al. demonstrated that *MYC* activation is the pivotal event that licenses AT2 cells to overcome lineage-intrinsic barriers and complete their reprogramming into bona fide SCLC, underscoring *MYC* as a central driver of neuroendocrine trans-differentiation (14). Aurora-kinase, *BET*, and *PIM2* inhibitors indirectly destabilize or down-regulate *MYC* and have demonstrated pre-clinical efficacy against *MYC*-amplified SCLC, providing a rational therapeutic entry point for this subset (73). These agents are therefore poised for investigation in T-SCLC harboring *MYC* amplification.

*SOX2*, a key guardian of oncogenic self-renewal, is recurrently amplified and over-expressed in both de-novo and transformed SCLC, making it a potential therapeutic target. As a transcription factor, *SOX2* is considered undruggable; however, proteasome inhibitors or RNA-interference–mediated silencing can indirectly down-regulate its expression and thereby enhance cisplatin sensitivity in SCLC (74).

*BCL-2*, an anti-apoptotic member of the *BCL-2* family, is frequently over-expressed in a subset of T-SCLC, conferring a survival advantage and chemo-resistance (75). Pre-clinical studies show that the *BCL-2* inhibitor venetoclax abrogates downstream survival signals, eliciting robust apoptosis in *BCL-2*-high SCLC cell lines and corresponding patient-derived xenografts, resulting in marked tumor growth suppression (76). Next-generation dual *BCL-2*/*BCL-xL* degraders—exemplified by PROTAC constructs—exhibit superior antitumor potency and an improved therapeutic index in pre-clinical models, positioning them as promising precision options for T-SCLC (77).

In *RB1*-deficient SCLC, pharmacologic inhibition of the SCF-Skp2 E3 ubiquitin ligase or Aurora B kinase has demonstrated marked antineoplastic efficacy, providing a rational therapeutic strategy for transformed tumors harboring biallelic *RB1* loss (78). Skp2 operates as a critical downstream effector of pRb; *RB1* loss derepresses Skp2, amplifying its oncogenic activity and accelerating tumorigenesis (56). Small-molecule Skp2 inhibitors robustly suppress tumor growth in both human and murine *RB1*-null SCLC models, exerting potent activity against primary lesions, liver metastases, and chemotherapy-refractory tumors, thereby offering a promising therapeutic avenue for this genomically defined subset (79). In addition, TP53- or RB1-deficient tumors may exhibit synthetic-lethal vulnerabilities to DNA-damage-response and cell-cycle checkpoint inhibitors, including ATR, CHK1, WEE1, PARP, and Aurora kinase inhibitors, although these strategies remain largely preclinical in T-SCLC.

## 5 Immunotherapeutic strategies

Immune-checkpoint inhibitors (ICIs) are integral to advanced NSCLC management; by interrupting *PD-1/PD-L1* or *CTLA-4* signaling they counteract tumor immune-evasion mechanisms and reinvigorate cytotoxic T-cell activity (80). Nevertheless, efficacy of ICIs in T-SCLC remains modest, attributable to a profoundly immunosuppressive microenvironment, persistently low tumor mutational burden (TMB), and down-regulated antigen-presentation machinery (20). In the series reported by Marcoux et al., 17 T-SCLC patients treated with *PD-1/PD-L1* monotherapy or combined with *CTLA-4* blockade derived no clinical benefit, the longest PFS being only 9 weeks (38). In contrast, a retrospective study of 47 patients with T-SCLC suggested potential benefit in selected patients. In this study, *PD-L1*–positive patients treated with atezolizumab plus platinum–etoposide ± bevacizumab achieved a median OS of 20.2 months compared with 7.9 months in those receiving chemotherapy alone, with an ORR of 73%. However, these findings should be interpreted with caution because of the retrospective design, limited sample size, potential selection bias, and lack of prospective validation (20).

## 6 Novel therapeutics

Several molecular drivers of SCLC progression have recently entered clinical translation. Accordingly, multiple novel agents are being evaluated in early-phase trials.

### 6.1 *DLL3*-directed targeted therapy

*DLL3* is a transmembrane protein highly expressed in SCLC and other neuroendocrine tumors, while its expression in normal adult tissues is limited, making it an attractive target for precision therapy. In T-SCLC, *DLL3* expression is frequently associated with acquisition of neuroendocrine features, providing a biological basis for its therapeutic relevance in T-SCLC (21, 81).

Several *DLL3*-targeted therapeutic strategies have been developed, including antibody–drug conjugates, bispecific T-cell engagers, cellular therapies, and radioimmunotherapy platforms (49). These approaches aim to eliminate *DLL3*-expressing tumor cells through cytotoxic payload delivery or immune cell redirection. Among these, tarlatamab, a *DLL3* × *CD3* bispecific T-cell engager, has demonstrated the most advanced clinical evidence (82). In a phase III trial (DeLLphi-304), tarlatamab significantly improved overall survival compared with standard chemotherapy in relapsed SCLC, supporting *DLL3* as a clinically actionable target in this disease (83).

However, most available evidence is derived from *de novo* SCLC, and data in T-SCLC remain limited. Given the potential for *DLL3* upregulation during neuroendocrine transformation, future studies should evaluate *DLL3* expression dynamics in paired pre- and post-transformation samples and determine its predictive value for therapy selection in T-SCLC (21) (Table 3).

**Table 3:** Summary of therapeutic strategies and clinical outcomes in transformed small-cell lung cancer.

Treatment strategy	Drug names/regimens	ORR	PFS	OS	Reference
Platinum–etoposide chemotherapy	Etoposide + cisplatin/carboplatin	44.4–54%	3.0–3.5 months	5.7 months to NR	(7, 36, 37, 39)
Taxane-based chemotherapy	Paclitaxel; docetaxel	0–50%	2.7 months to NR	NR	(36, 52)
Chemotherapy combined with EGFR-TKI	Platinum-based chemotherapy or other chemotherapy + EGFR-TKI	43.8–44.4%	5.2–7.2 months	14.8–38.9 months	(7, 46, 52, 55)
Chemotherapy alone comparator groups	Platinum-based chemotherapy or other chemotherapy	16.7–37.5%	3.0–3.7 months	13.0–34.4 months	(7, 55, 62)
Anti-angiogenic therapy	Anlotinib ± chemotherapy / EGFR-TKI / immunotherapy	NR	NR	15.1 months	(55, 64, 65)
Immune-checkpoint inhibitor therapy	PD-1/PD-L1 inhibitor ± CTLA-4 inhibitor	No clinical benefit reported	Longest PFS: 9 weeks	NR	(20, 36)
PD-L1 inhibitor-based chemo-immunotherapy	Atezolizumab + platinum–etoposide ± bevacizumab	73%	NR	20.2 months	(19, 20)
ALK-directed strategy	Platinum-based chemotherapy ± ALK-TKI	50–66%	3–4 months	6–12 months	(41, 42)
DLL3-directed therapy	Tarlatamab and other DLL3-directed agents	NR	NR	NR	(20, 49, 81–83)

ORR, objective response rate; PFS, progression-free survival; OS, overall survival; NR, not reported; SCLC, small-cell lung cancer; T-SCLC, transformed small-cell lung cancer; EGFR-TKI, epidermal growth factor receptor tyrosine kinase inhibitor; ICI, immune-checkpoint inhibitor; DLL3, delta-like ligand 3. Most available evidence for transformed SCLC is derived from retrospective cohorts, pooled analyses, small case series, or case reports. Therefore, direct comparisons across studies should be interpreted with caution because of heterogeneity in baseline histology, prior treatment exposure, diagnostic criteria for transformation, molecular profiling, and post-transformation treatment regimens.

### 6.2 Epigenetic-targeting therapeutics

Epigenetic therapies in SCLC have primarily focused on DNA methylation and histone modifications (84). Among these, *EZH2* inhibitors—including valemetostat, tazemetostat, and HM97662—are the most mature in SCLC; they suppress H3K27 trimethylation, thereby restoring MHC-I and CCL2 expression, augmenting immune recognition, and reversing chemo-resistance. Reduced *EZH2* expression correlates with heightened sensitivity to immune-checkpoint blockade and cisplatin, an observation that has prompted ongoing clinical trials (NCT03460977, NCT03525795, NCT04104776). Desai et al. (84) initiated the first epigenetic-chemo-immunotherapy triplet (tazemetostat + topotecan + pembrolizumab) for SCLC in 2024; safety and efficacy results are awaited. Additional epigenetic agents under evaluation include *LSD1* (*KDM1A*) inhibitors (85), DNA methyltransferase inhibitors (*DNMTi*) such as decitabine and azacitidine, histone deacetylase inhibitors (*HDACi*), and BAP1 inhibitors, although all remain in early-phase trials or pre-clinical models (56).

## 7 Discussion

T-SCLC can arise from diverse NSCLC subtypes with distinct molecular underpinnings (13). Although T-SCLC often retains baseline NSCLC-associated mutations, its biological behavior and molecular profile become closer to those of de novo SCLC, while clinical outcomes are generally worse (86). Moreover, treatments administered before transformation usually fail to control the transformed disease, highlighting the need for more accurate diagnosis and optimized therapeutic strategies (8, 21).

A central challenge in T-SCLC is accurate diagnosis. In clinical practice, apparent transformation may reflect either true lineage plasticity under therapeutic pressure or the expansion of an initially under-sampled combined SCLC component (46, 47). This distinction is clinically important but often difficult, especially when baseline tissue is limited (23). Therefore, repeat biopsy remains essential when patients develop rapid disease progression, atypical metastatic patterns, increasing neuroendocrine markers, or unexpected resistance to targeted therapy or immunotherapy (87, 88). Histologic reassessment should ideally be combined with neuroendocrine immunohistochemistry and molecular profiling to confirm clonal relatedness between the initial NSCLC and T-SCLC components. In this context, *TP53*/*RB1* co-inactivation, particularly in *EGFR*-mutant lung adenocarcinoma, represents one of the most consistent molecular risk signatures for transformation (89, 90). Additional genomic features, such as whole-genome doubling and increased CNV burden, may further refine risk stratification, although their clinical utility still requires prospective validation (24, 48, 62).

Surveillance strategies are also important because transformation is often recognized only after overt clinical progression (34). Serum neuroendocrine markers, including NSE, Pro-GRP, may provide supportive clues but lack sufficient specificity to establish the diagnosis independently (30). Liquid biopsy approaches, including ctDNA and circulating tumor cells, may allow longitudinal monitoring of molecular evolution and emerging resistant subclones (17, 91). Nevertheless, liquid biopsy should currently be viewed as a complementary tool for risk assessment and early warning rather than a substitute for tissue confirmation (17). Future surveillance models should combine clinical behavior, serum markers, imaging features, and serial molecular profiling to identify high-risk patients before extensive disease progression occurs (17, 91).

Therapeutically, platinum-etoposide chemotherapy remains the backbone of treatment after transformation. Available retrospective data suggest that this regimen can induce initial tumor responses, but the duration of benefit is usually short, and disease progression commonly occurs within months (8, 19). This indicates that chemotherapy alone is insufficient for durable disease control. In patients retaining *EGFR* mutations, continuation of *EGFR-TKI* in combination with chemotherapy may improve response rate and progression-free survival compared with chemotherapy alone, but an overall survival advantage has not been consistently demonstrated (8, 62). These findings suggest that transformed tumors may retain partial dependence on the original driver pathway while simultaneously acquiring SCLC-like biology, resulting in reduced sensitivity to driver-targeted monotherapy. Therefore, continued driver-targeted therapy should be considered selectively rather than routinely, preferably guided by post-transformation molecular profiling (8, 19).

Anti-angiogenic therapy may represent a useful adjunctive option in T-SCLC. Among available agents, anlotinib has demonstrated modest activity in relapsed SCLC and in limited retrospective studies of transformed disease, particularly in later-line settings or when combined with chemotherapy or other systemic therapies (64, 92). However, the evidence remains preliminary and is constrained by retrospective design and small patient cohorts. Therefore, its use should be considered on an individual basis, and its definitive role in T-SCLC requires validation in prospective clinical studies (92, 93). The role of immunotherapy in T-SCLC is similarly limited and remains uncertain. Immune-checkpoint inhibitor monotherapy has shown minimal activity, likely reflecting the immunologically “cold” phenotype of transformed tumors (8, 21). Combination chemo-immunotherapy may provide benefit in selected subgroups, but current evidence is derived mainly from retrospective analyses and is not sufficient to support routine application (8, 20, 21). Biomarkers capable of identifying immunotherapy-responsive patients remain an unmet need in this setting (20). Several emerging molecular targets may expand future treatment options for T-SCLC. *DLL3* is of particular interest due to its association with neuroendocrine differentiation and its increased expression following transformation in some cases, making it a promising target for antibody–drug conjugates and bispecific T-cell engager strategies (21, 49, 83). Additional candidates, including *MYC*, *SOX2*, *BCL-2*, *RB1*-related vulnerabilities, and the *PI3K*/*AKT*/*mTOR* pathway, are biologically relevant but remain largely at the preclinical or early clinical stage (14, 56, 77). Similarly, epigenetic regulators such as *EZH2*, *LSD1*, *DNMT*, and *HDAC* represent potential therapeutic avenues, although most supporting evidence is currently derived from *de novo* SCLC rather than transformed disease. Further studies are required to determine their clinical relevance in T-SCLC (56, 84).

Future studies should focus on translating emerging molecular advances into precision treatment strategies for T-SCLC. Given the rarity of this disease and the current reliance on retrospective evidence, prospective studies incorporating standardized repeat biopsy, longitudinal molecular profiling, and biomarker-guided treatment stratification are urgently needed (8). Whenever feasible, repeat biopsy should prioritize fresh tumor tissue for transcriptomic, proteomic, metabolomic, single-cell, spatial, and integrated multi-omics analyses, while liquid biopsy may be expanded beyond ctDNA to include epigenomic profiling, circulating RNA, and longitudinal monitoring of tumor and immune evolution. Biomarker-driven basket and umbrella trials may also provide efficient approaches for evaluating targeted therapies in molecularly defined T-SCLC subgroups (11). Comparative analysis of paired pre- and post-transformation samples, together with single-cell and spatial transcriptomic approaches, may identify mechanisms of lineage plasticity, therapeutic resistance, and novel actionable targets (94). In parallel, liquid biopsy technologies, including ctDNA and circulating tumor cells, may enable real-time monitoring of molecular evolution and treatment response (17). Although platinum-based chemotherapy remains the therapeutic backbone, future research should clarify which patients may benefit from continued driver-targeted therapy, anti-angiogenic agents, chemo-immunotherapy, *DLL3*-targeted therapies, epigenetic modulators, or rational combination strategies (8, 83). Ultimately, integrating multi-omics profiling with clinical outcomes may facilitate biomarker-driven patient stratification and advance T-SCLC management from empirical treatment toward personalized, response-adapted therapy (12, 56).

## 8 Limitations

The evidence base for T-SCLC consists mainly of small retrospective cohorts with inherent selection and reporting biases (8, 21). Diagnostic criteria for histologic transformation were not standardized across studies, with variable requirements for morphologic assessment, immunohistochemistry (synaptophysin, chromogranin, *CD56*, *TTF-1*, *Ki-67*), and molecular confirmation of clonal relatedness. In particular, some reported cases may not have fully excluded pre-existing combined SCLC that was missed because of limited baseline biopsy sampling, particularly among cases emerging after chemotherapy or immune-checkpoint inhibitor therapy. Treatment heterogeneity—encompassing platinum agent selection (cisplatin versus carboplatin), *EGFR-TKI* generation, radiotherapy application, and immunotherapy timing—precludes definitive efficacy comparisons. Molecular characterization was incomplete; while *RB1*/*TP53* co-alteration is increasingly recognized as a transformation predictor, comprehensive profiling of *PI3K*-*AKT* pathway activation, *MYC* family amplification, and *DLL3* expression status was not uniformly reported, limiting predictive biomarker development. Because this was a narrative review, we did not perform a systematic risk-of-bias assessment or GRADE evaluation. Language restrictions and the exclusion of conference abstracts may have introduced publication bias. Finally, the median post-transformation survival of approximately 6 months suggests that observed treatment outcomes may be confounded by patient selection, performance status preservation, and lead-time bias rather than true treatment effects (95).
