# Supplementary material for: Advanced and underlying therapeutic strategies in transformed small cell lung cancer
Source: Front Med (Lausanne). 2026 Aug 27;13:1865050. doi: 10.3389/fmed.2026.1865050 (PMC13560167; doi:10.3389/fmed.2026.1865050)
Supplement: Supplementary file 1 [file Supplementary_file_1.docx]

**PubMed Search Strategy**

(

("non-small-cell lung cancer"[Title/Abstract] OR "non-small cell lung cancer"[Title/Abstract] OR NSCLC[Title/Abstract] OR "lung adenocarcinoma"[Title/Abstract])

AND

("small-cell lung cancer"[Title/Abstract] OR "small cell lung cancer"[Title/Abstract] OR SCLC[Title/Abstract])

AND

(transformation[Title/Abstract] OR "histologic transformation"[Title/Abstract] OR "histological transformation"[Title/Abstract] OR "small-cell transformation"[Title/Abstract] OR "small cell transformation"[Title/Abstract] OR "neuroendocrine transformation"[Title/Abstract])

AND

(

"lineage plasticity"[Title/Abstract]

OR "clonal evolution"[Title/Abstract]

OR "clonal selection"[Title/Abstract]

OR TP53[Title/Abstract]

OR RB1[Title/Abstract]

OR "epigenetic"[Title/Abstract]

OR "tumor microenvironment"[Title/Abstract]

OR biomarker*[Title/Abstract]

OR "molecular profiling"[Title/Abstract]

OR "liquid biopsy"[Title/Abstract]

OR ctDNA[Title/Abstract]

OR "circulating tumor DNA"[Title/Abstract]

OR NSE[Title/Abstract]

OR "Pro-GRP"[Title/Abstract]

OR chemotherapy[Title/Abstract]

OR "platinum-etoposide"[Title/Abstract]

OR "platinum etoposide"[Title/Abstract]

OR EGFR-TKI[Title/Abstract]

OR "EGFR inhibitor"[Title/Abstract]

OR "immune checkpoint inhibitor"[Title/Abstract]

OR immunotherapy[Title/Abstract]

OR "anti-angiogenic"[Title/Abstract]

OR anlotinib[Title/Abstract]

OR DLL3[Title/Abstract]

OR "molecular targeted therapy"[Title/Abstract]

)

)

AND

("2006/01/01"[Date - Publication] : "2025/06/1"[Date - Publication])
